# Supplementary material for: MetaRibo-Seq measures translation in microbiomes
Source: Nat Commun. 2020 Jun 29;11:3268. doi: 10.1038/s41467-020-17081-z (PMC7324362; doi:10.1038/s41467-020-17081-z)
Supplement: Supplementary file 10 — Supplementary Data 7 [file 41467_2020_17081_MOESM10_ESM.zip › File2/Confidence_High_Taxonomy/182006_out.krona.html]

Javascript must be enabled to view this page.

members
magnitude
magnitudeUnassigned
count
unassigned
taxon
rank

182006\_out

60

60
superkingdom
2

976
phylum
60

200643
class
60

60
order
171549

60
family
815

5

SRS013800\_contig\_number\_15366SRS015579\_contig\_number\_25810SRS048870\_contig\_number\_15698SRS1041039\_contig\_number\_contig-100\_3323.148548SRS1041137\_contig\_number\_contig-100\_3115.178536
genus
816
60

species

SRS024663\_contig\_number\_8335
818
1


SRS098717\_contig\_number\_12709
species
817
1

457391

SRS043001\_contig\_number\_25
species
1

2292935

SRS014923\_contig\_number\_46638SRS050925\_contig\_number\_3446
species
2

7
species

SRS012849\_contig\_number\_contig-100\_3289.178952SRS016381\_contig\_number\_275SRS023715\_contig\_number\_11517SRS053649\_contig\_number\_19088SRS054590\_contig\_number\_348SRS147139\_contig\_number\_9630SRS148511\_contig\_number\_19637
821

1
species

SRS024435\_contig\_number\_25096
338188

41
357276
species

SRS012273\_contig\_number\_4876SRS013098\_contig\_number\_46374SRS013215\_contig\_number\_1458SRS013476\_contig\_number\_33738SRS013940\_contig\_number\_4788SRS015890\_contig\_number\_481SRS016018\_contig\_number\_3488SRS018049\_contig\_number\_229SRS018541\_contig\_number\_1694SRS018575\_contig\_number\_1332SRS018984\_contig\_number\_7552SRS019685\_contig\_number\_41491SRS022071\_contig\_number\_33893SRS023346\_contig\_number\_1047SRS024132\_contig\_number\_20501SRS024549\_contig\_number\_3288SRS042628\_contig\_number\_31732SRS045195\_contig\_number\_6206SRS046717\_contig\_number\_8249SRS048981\_contig\_number\_1659SRS052078\_contig\_number\_2092SRS056519\_contig\_number\_6132SRS057717\_contig\_number\_2961SRS058070\_contig\_number\_28320SRS058770\_contig\_number\_22263SRS075021\_contig\_number\_1239SRS075341\_contig\_number\_8771SRS077024\_contig\_number\_10032SRS078419\_contig\_number\_21090SRS098655\_contig\_number\_3174SRS101268\_contig\_number\_729SRS1041112\_contig\_number\_5288SRS104636\_contig\_number\_147SRS1055034\_contig\_number\_10603SRS140645\_contig\_number\_631SRS142712\_contig\_number\_2104SRS143523\_contig\_number\_15582SRS143598\_contig\_number\_12584SRS148874\_contig\_number\_11214SRS149784\_contig\_number\_26401SRS149879\_contig\_number\_25345

1
species

SRS019286\_contig\_number\_5176
371601
